# England and Her Christmas

**Published:** 1893-12-23

**Authors:** 


### The Hospital

An Institutional Journal of

Science, Medicine, Hygiene, Nursing, and News.

*For Hospitals, Asylums, Infirmaries, Dispensaries, Medical Practitioners, Students, and Nurses.*

Vol. XV.—No. 378.] [Dec. 23, 1893.

### England and her Christmas.

A boy of four was told a day or two ago that he must hang up his stocking on Christmas Eve and Santa Claus would fill it with beautiful presents. "Mother will fill it," answered the infantine critic of the *fin de siécle* The child had his doubts about Santa Claus, but his faith in his mother was absolute. With grown-up people, as with the young, the belief in myth and miracle is lessening year by year; but as this faith diminishes there is a steady growth of confidence and trust in right-minded human beings, in high moral principle, intelligent religion, and the divine-human brotherhood, lived, expounded, and died for by the founder of Christianity. The loss of faith in myth and merely sentimental worship is nearly all gain. The growth in intelligent comprehension of great principles, of the sense of obligation to love and practice "the highest when we see it," and in appreciation of the permanently enduring value of such an object lesson as was given to the world by the divine-human nearly nineteen hundred years ago is a gain to intellect, to morals, to behaviour, and to brotherhood which is immeasurably great.

The present is a time of depression: is it also a time of real national advancement? It is, and it is not. To the weak it is not. The weak never advance. They may be carried forward by the stream of time tendency, but they do not advance. The strong advance; and they advance all the more after a time of forced repression, and limitation by unyielding environment. Many people cannot take a measure of the modern world at all. They see, or think they see, the wild billows of socialism and anarchism surging on every side. They do not know whether any barque of politics, economics, or even morals, will be found strong enough to outride the storm. The true British heart, fortified by convictions as clear as Shakespeare's, as old as Augustine's, as human and brotherly as King Alfred's of undying memory, feels no fear. The British races were made to rule the storm; they have been developed for the purpose of comprehending and propagating a noble humanity, a humanity founded in justice and love, of which the first Christmas was the inauguration day, and every succeeding Christmas has been the joyful anniversary.

The present time of depression is a time for resolve; and, above all, it is a time for duty—for the special duty of the season and the moment. Our national history has made Christmas for us a halting place. It is a season in which the whole nation, great and small, draws rein. It is a season in which the prosperous and the happy have been wont to open wide a generous heart, and to share liberally and joyously with the poor. It is a season in which the poor, ever since the days when Queen Elizabeth defied the Armada, ever since those earlier days when Harold disputed the field of Hastings with the Norman, ever since those still remoter times when the Saxons and the ancient Britons, at the bidding of the pious Austin, abandoned their superstitions and accepted the reasonable religion whose symbol is a cross—ever since, in short, the true beginning of our national life, the poor have been for a brief period lifted out of their poverty, and made welcome to the abundance, the brightness, and the freeheartedness of the rich. *Noblesse oblige!* We as a race and a nation cannot cut ourselves off from our past. We have been a strong people, we have been a brave people, we have been a free-handed people; we have struggled through, and defied, and risen above times of stress and adversity in the past: we must still be a strong people, a brave people, a free-handed people; and we must rise above this time of strain and depression by the great qualities of courage, practical sense, and invincible resolution which have carried us through all past times, and placed us to day in the vanguard of the world. Above all, in our time of stress and strain we must look with eyes of brotherly kindness on those upon whom the stress and strain fall hardest, upon the real poor, the sick, the suffering, and the helpless; and with a courage and a self-denial which are the true marks of greatness we must, because the times are bad, do greater and nobler deeds of generosity and charity than ever we have done before.

Our appeal for the hospitals this Christmas is not merely to the philanthropic—it is to the nation; to the great nation, a portion of whose history we have just glanced at. In ancient times our nation was great; in modern times it is greater still. Greatest of all its greatnesses are its splendid charities. The solid walls of our hospitals, their noble proportions, their magnificent revenues, and the divine and incomparable work which they do throughout the year are proofs of our national worthiness which no unfriendly critic can dispute, and no cynic can diminish by a hairsbreadth. So long as we do this great work, and do it so greatly, so generously, and so perseveringly, the world may laugh us to scorn, but it cannot lessen by a single feather's weight the actual value of our national character. These are the things we have always done and are now doing. Let our critics check the flow of their criticism and do better work than ours.

Last Christmas the hospitals, though we did not then know it or even dream of it, were close upon the discovery of a deficit of £171,881 after the year's working. The discovery, when it came, was a terrible blow to those who had set their hearts upon the maintenance and upward progress of the hospitals. What may be the amount of the deficit which will be brought to light at the close of the present year it is impossible at present to guess. It will probably be disastrously great. Unless, indeed, some unusual effort is made, it will certainly be disastrously great. Have we as a nation the power to make a supreme effort on behalf of the hospitals at the present crisis? The question will raise a smile. The power? We have the power to rebuild all the hospitals from their foundations, and to re-endow them if they required it. We are passing through a period of depression, it is said. Truly; but of what kind is the depression? It is of a kind which is strictly temporary, and which prevents fortunes being made in a few years. But is the country seriously unprosperous? Are any of us poor? Will the least prosperous of us do without our Christmas turkeys, our wines, our holidays, or the presents of our friends? The country is rich. Never did it contain so much realised wealth as now. We are not desirous of under-estimating for a moment the real nature of the depression which is upon us. But we say that it will all have been forgotten five years hence, and not one of us will be one whit the less happy, though many of us may be by many degrees the better for having passed through it.

"Courage" is our motto; "courage and duty done." The brave man faces his difficulties; the honest man meets his obligations. Our hospitals are in difficulties; we must face them. We have taught the poor to depend upon us to supply them with the best medical skill of the times when they are sick. These are obligations which we have voluntarily contracted. We must meet them. True, they are obligations which know neither lawyer, nor parchment, nor stamp, and, therefore, we may evade them if we will. But we are a people who hate dishonour. Because these obligations know neither lawyer, nor parchment, nor stamp—for that very reason we will meet them all the more. The one great obligation we have now to meet is a deficit—a terrible, and, to the hospitals, an overwhelming deficit. We must meet the deficit with our highest courage, and we must annihilate it. No less than £200,000 ought to be raised this Christmas; a tough battle, but an honourable, if it be faced bravely and fought successfully. How many Davids are there at this Christmas season who will play the hero's part and vanquish for the nation this monstrous Goliath of hospital debt?

